# Design and Fabrication of a Three-Dimensional Artificial Compound Eye Using Two-Photon Polymerization

**DOI:** 10.3390/mi9070336

**Published:** 2018-07-02

**Authors:** Jieqiong Lin, Yudi Kan, Xian Jing, Mingming Lu

**Affiliations:** Key Laboratory of Micro/Nano and Ultra-Precision Manufacturing, School of Mechatronic Engineering, Changchun University of Technology, Changchun 130012, China; linjieqiong@ccut.edu.cn (J.L.); kanyudi@outlook.com (Y.K.); lumm@ccut.edu.cn (M.L.)

**Keywords:** compound eye, microlens, two-photon polymerization, ray tracing

## Abstract

Microlens arrays have been widely used in the fields of micro-optics because of the advantages of their high diffraction efficiency, high fill factor, and wide operating band. However, the microlens array still has problems with its smaller field of view (FOV) and lower utilization of light energy. In this paper, a 3D compound eye system consisting of a microlens array and a pinhole array was designed according to the optical principle of insect compound eye. The artificial compound eye structure was processed in two-photon polymerization processing technology. Ray tracing and optical system simulation of the designed artificial compound eye structure were performed. The results showed that the artificial compound eye structure had a wider FOV and higher light energy utilization than a conventional 2D microlens array. This thesis may lay a theoretical foundation for the structural optimization design of microlens arrays.

## 1. Introduction

Micromachines can provide interfaces in the fields of biology and optics, medicine, fluidics, etc. [1,2,3]. A microlens array is composed of microlenses arranged in a plane with a certain period [4]; its appearance marks the rapid development of modern optics into miniaturization and integration. In recent years, microlens arrays have been widely used in the field of micro-optics, such as with laser beam homogenizer [5], Hartmann–Shack wavefront sensors [6], 3D integral imaging systems [7], optical fiber coupling [8,9], fluorescence detection [10,11], medical diagnosis [12,13], and focused-beam dipole traps [14], due to the advantages of its high diffraction efficiency, high fill factor, and wide operating band. Optical information can be imaged without interference between the array elements of the microlens array. However, the entire array structure has a smaller FOV and lower utilization of light energy due to a lack of three-dimensional micro-optical surfaces and sensitometric beams in the structure compared with compound eyes in nature.

The compound eye in nature has many advantages, such as a wide FOV [15], high sensitivity characteristics, and a highly efficient utilization of light energy. For decades, the compound eye has been widely investigated in various research fields. In this paper, the structure of a 2D microlens array was optimized. A 3D curved compound eye structure composed of a micro-lens array and pinhole array was designed according to the optical principle of a bionic compound eye [16,17,18]. A microlens array on a curved surface was designed to improve the utilization of light energy and enhance the convergence of light at the edges of the compound eye. The pinholes were designed to play a role as a sensitometric beam to get rid of the cross-talk of optical information during imaging process and expand the FOV.

At present, there are already many technologies that can achieve compound eye processing, such as the femtosecond-laser-enhanced local wet etching method [19], transparent grapheme electrode [20], and ultraprecision diamond turning process [21]. In recent years, two-photon polymerization (TPP) has been widely considered as a very promising method for processing 3D micro/nano structures. The microstructure of the bionic compound eye in this design was fabricated in femtosecond laser two-photon polymerization technology. Compared with other technologies for processing compound eye structures, the advantages of two-photon polymerization are that its processing environment and parameters are easy to control; it adopts layered additive manufacturing (AM) methods in order to avoid assembly errors; and it can realize micron sample processing.

## 2. Design of the Compound Eye System

**Figure 1:**
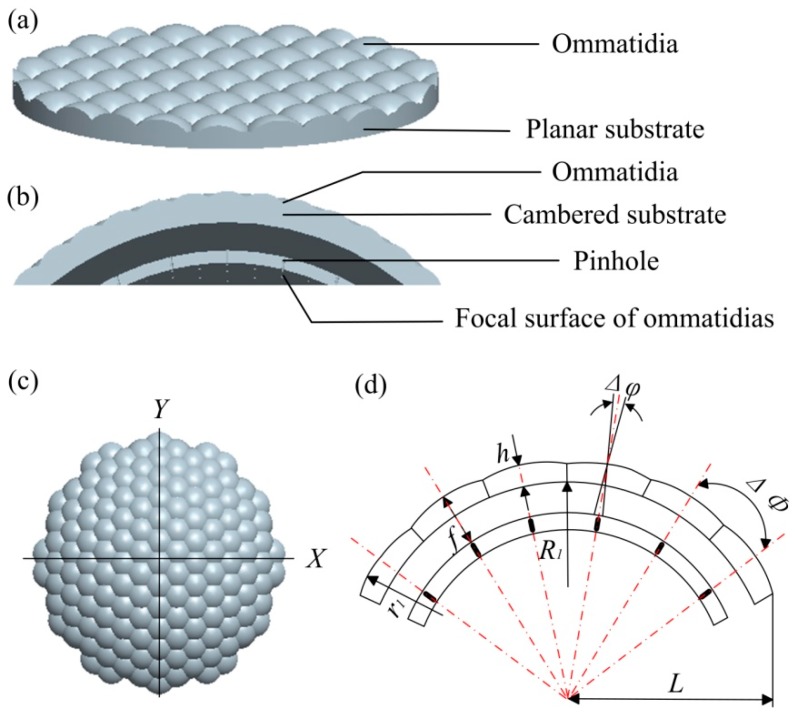
Original model of the microlens and compound eye: (**a**) the 3D model of ordinary curved microlens arrays; (**b**) the cross section of the compound eye; (**c**) the top view of compound eye; (**d**) the 2D structure of compound eye along the *X* direction in Figure 1c.

An ordinary microlens array is obtained by microlenses being arrayed with a definite period on a planar substrate. The structure is single without three-dimensional micro-optical surfaces and a sensitometric beam. The three-dimensional model is shown in Figure 1a. In this paper, a new microlens array composed of a micro-lens array and pinhole array was designed according to the optical imaging and design principles of bionic compound eye. The microlens array on a cambered substrate was designed to converge incident lights from different directions onto the imaging plane and improve the utilization of light energy. The pinhole array layer, which was formed by stitching the focal plane of each ommatidia, was added inside the three-dimensional space structure. The pinhole was required to be consistent with the optical axis of its corresponding microlens so that it accurately guided the light refracted by the ommatidia onto the imaging plane. The pinhole design reduced the possibility of cross-tracking during imaging and expanded the angle of view. The cross section of the compound eye structure designed in this paper is shown in Figure 1b.

Each ommatidia was designed as a spherical hexagonal structure in order to make its topography and optical properties more closely emulate the insect compound eye. A uniform hexagonal array was adopted which was inspired by the compact structure of the honeycomb and its non-collapsibility. The advantage of this hexagonal structure was that each ommatidia formed a “cluster eye” with its six surrounding channels. The top view of the 3D model is shown in Figure 1c.

The diameter (*d*_1_) of each ommatidia in insect compound eye is within the range of 10–50 μm. A compound eye with a diameter of 10 μm in each ommatidia was taken as an example in this paper, and a 30 μm × 30 μm × 15 μm three-dimensional compound eye structure was designed. That is, the sectional radius *L* of curved substrate is 30 μm, the overall height of structure is 15 μm, the curved base is a spherical surface with a radius of curvature *R*_1_ = 50 μm. The two-dimensional plane of the structure along the *X* direction is shown in Figure 1d.

The focal length *f* of each ommatidia can be obtained from the focal length formula of the spherical lens:

(1) $$f=\frac{nr_{1}R_{1}}{(n-1)\left[n(R_{1}-r_{1})+(n-1)h\right]}$$

where *n* is the refractive index of microlens, and the refractive index of Ormocer photoresist is 1.5; *r*_1_ is the radius of ommatidia; *h* is the central thickness of spherical ommatidia, *h* = 5 μm. Because each ommatidia is a convex lens, *r*_1_ takes a positive value and *R*_2_ takes a negative value.

The angular resolution of the compound eye imaging system is defined by the angle Δ*Φ* between adjacent optical axes. The angular resolution is one of the most important factors for expanding the FOV and affecting the optical sensitivity of the compound eye, which is obtained from the geometry:

(2) $$\Delta \Phi =2\arctan\frac{l}{2R_{1}}$$

where *l* is the distance between adjacent ommatidias.

The radius of the pinhole cross-section should be approximately equal to the size of the lens’ point spread function (PSF) [22]:

(3) $$r_{2}=1.22\lambda F$$

where *λ* is the wavelength of incident light and its value is determined by the maximum wavelength in the He-Ne laser, thus *λ* = 0.6328 μm. *F* is working as *F*/*#*, and *F*/*#* = *f*/*d’*, due to the ommatidias being arranged in a hexagonal array and closely connected; *d’* < *d*_1_, *d’* = *l,* thus *F* = *f*/*l.*

The optical axis of each ommatidia points to different angles in the object space. The light received by each ommatidia only comes from the corresponding object space angle Δ*φ*. Δ*φ* can be obtained by the geometric relationship:

(4) $$\Delta \varphi =2\arctan\frac{r_{2}}{f}$$

**Figure 2:**
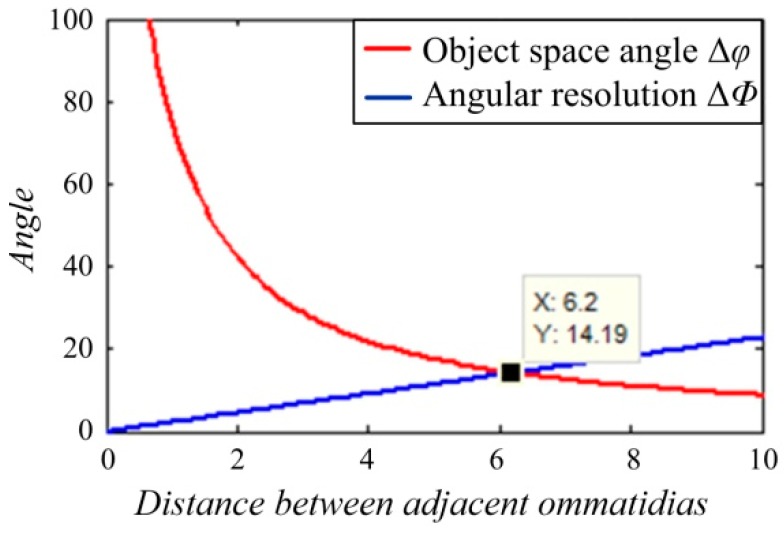
The curves of object space angle Δ*φ* and doubled angular resolution Δ*Φ* as the change of distance *l* between adjacent ommatidias.

The accessional constraints of Δ*φ* = 2Δ*Φ* must be considered to ensure that two adjacent channels can accurately distinguish a pair of diffractive ripples alternating between light and dark. The curves of Δ*φ* and 2Δ*Φ* were plotted as the change of parameter *l*, as shown in Figure 2.

The *X* value in Figure 2 means the optimal value of the distance between adjacent ommatidias; that is, *l* = 6.2.

**Figure 3:**
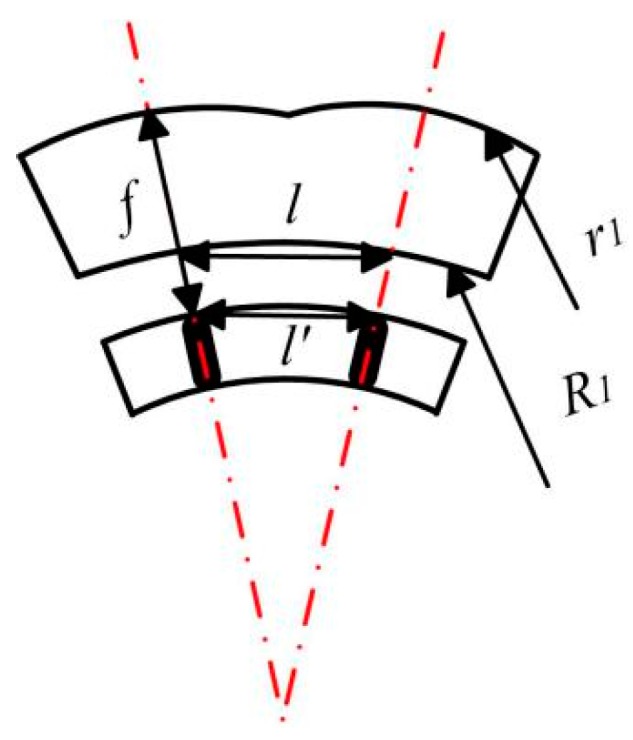
The positional relationship of adjacent ommatidias.

The positional relationship of adjacent ommatidias is shown in Figure 3. *l’* is the distance between adjacent pinholes, according to the similar triangle which can be calculated as follows:

(5) $$\frac{l}{l^{\prime }}=\frac{R_{1}}{R_{1}+r_{1}-f}$$

The pitch difference of the pinhole array and the ommatidias array for a lens is given from Δ*l* = *l*−*l’*. The FOV (*ψ*) of one ommatidia is calculated from

(6) $$\psi =2\arctan\frac{\Delta l}{2f}$$

The compound eye’s FOV in the *X* direction in Figure 1c is *Ψ*_1_:

(7) $$\Psi_{1}=(2N-1)\psi +2(N-1)\Delta \Phi$$

The compound eye’s FOV in the *Y* direction in Figure 1c is *Ψ*_2_:

(8) $$\Psi_{2}=(2N-1)\psi +2\left(N-1\right)\Delta \Phi \cos30{}^{\circ}$$

where *N* is the number of layers in the microlens array. By sorting out the above formulas, the optimization function of the FOV is obtained:

(9) $$\{\begin{array}{c} \Psi_{1}\;=\;2\left(2N-1\right)\arctan\left[\frac{l\left(f-r_{1}\right)}{2f}\right]+4\left(N-1\right)\arctan\frac{1.22\lambda }{l}\\ \Psi_{2}\;=\;2\left(2N-1\right)\arctan\left[\frac{l\left(f-r_{1}\right)}{2f}\right]+3.464\left(N-1\right)\arctan\frac{1.22\lambda }{l} \end{array}$$

The entire FOV of the compound eye is *Ψ*_1_ × *Ψ*_2_. The theoretical FOV of the compound eye designed can reach 107.48° × 97.97° by calculation.

A commercial sensitometric beam is added during planar microlens array imaging, and the angle of view of each microlens is also Δ*φ*. However, because there is no angular sensitivity in the structure, the FOV in one dimension is *Ψ’*:

(10) $$\psi^{\prime }=(2N-1)\psi$$

In addition, the entire FOV of planar microlens array is *Ψ’* × *Ψ’* = 36.52° × 36.52°. Obviously, the FOV is less than one compound eye.

## 3. Fabrication of the Compound Eye System

Femtosecond laser two-photon polymerization technology was used to fabricate the microstructure of the bionic compound eye in this paper. The fundamental difference from other micromachining processes is that the entire optical structure was created during a machining process by layered additive manufacturing in order to avoid assembly errors. Ormocer [23,24], which is an inorganic–organic hybrid material, was used as the compound eye preparation material.

The bottom of the compound eye should be attached to a coverslip. A coverslip uniformly coated with Ormocer photoresist was prebaked at 90 °C for 3 min before exposure. A 800 nm wavelength, 100 fs pulse width, mode-locked Ti:sapphire oscillator with a repetition frequency of 80 MHz was used in this experiment. The laser power measured by a power meter was 48 mW. A layer thickness of 0.5 µm was used to fabricate the microstructure of compound eye. The coverslip with polymer structure was post-baked at 130 °C for 10 min after exposure. Then, a polymeric structure was produced in Ormocer.

**Figure 4:**
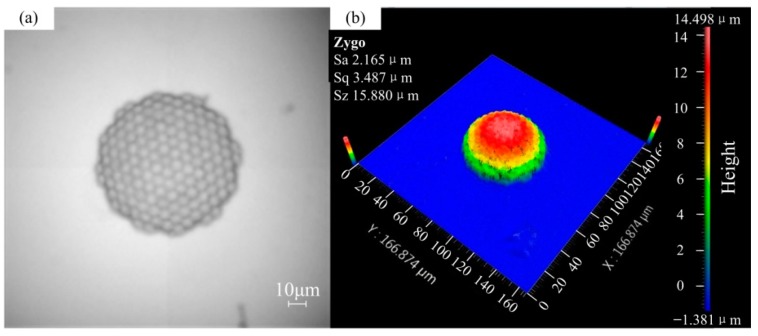
The test result of the designed compound eye: (**a**) Optical detection of the artificial compound eye; (**b**) Size measurement of the artificial compound eye.

After the sample was obtained, the coverslip with polymer structure was dipped in an OrmoDev developer (micro resist technology GmbH, Berlin, Germany) for 2–3 min, rinsed in alcohol to remove unexposed photoresist, and hard-baked at 150 °C for 3 h. The sample obtained was tested by NEWVIEW 600 optical profiler (Zygo Corporation, Middlefield, CT, USA). The results are shown in Figure 4. The physical structure of the artificial compound eye and each ommatidia are clearly seen in Figure 4. This showed that the sample had a good light transmission and high quality surface topography. The size measurement of the artificial compound eye is shown in Figure 4b. The entire compound eye structure has a total of 140 optical channels and meets our design requirements by measuring each dimension.

## 4. Optical Simulation and Discussion

**Figure 5:**
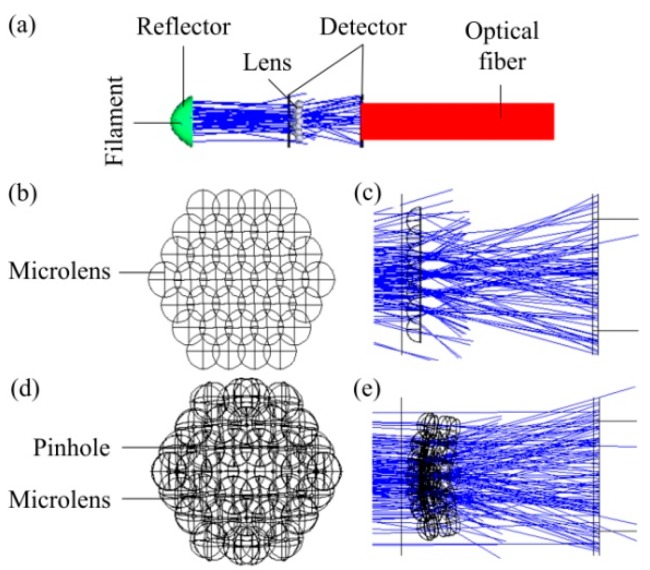
Simulated optical system: (**a**) Structural view of the virtual optical system; (**b**) Spliced microlens array structure view; (**c**) The ray tracing result of the planar microlens array; (**d**) Spliced compound eye structure view; (**e**) The ray tracing result of the compound eye.

In order to understand the light condensing ability and light energy utilization of the microlens array, the optical system was simulated in a non-sequential mode of the Zemax software (2009, Zemax, LLC, Kirkland, WA, USA) based on an optical path design in an optical system. Parallel light consisting of filaments and mirrors was used as virtual light source. Rectangular volume Alesis digital audio tape (ADAT) optical fiber was used as a signal relay. The detector was a 30 μm × 30 μm rectangle. A four-layer microlens array was token as an example to perform lens structure stitching in this article. The entire virtual optical system is shown in Figure 5a. A microlens array spliced into a plane in the form of a regular hexagon is used as a lens in the system. The spliced microlens array structure view is shown in Figure 5b. The ray tracing result of the planar microlens array is shown in Figure 5c. A two-layer regular hexagonal surface consisting of a microlens array and a cylindrical lens array with a central pinhole is used as the lens in the system. The spliced compound eye structure view is shown in Figure 5d. The ray tracing result of the compound eye is shown in Figure 5e. Obviously, the edge of the compound eye can accurately converge light on detector. Whereas a two-plane microlens array cannot. The degree of light deflection at the edge of a compound eye is stronger than that of a flat microlens array. In addition, the light in Figure 5c is directly received by the detector after being converged by the micro lens. However, in Figure 5e, after the light is converged by ommatidias, it is again converged by pinholes to the central optical axis of the compound eye and guided to the detector. A conclusion can be obtained that the compound eye structure designed in this paper has a wider FOV than that of an ordinary microlens array.

**Figure 6:**
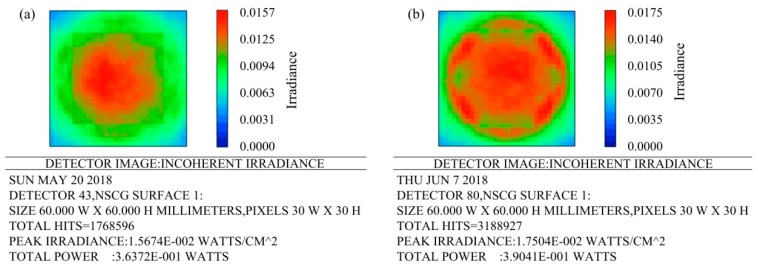
Light intensity distribution: (**a**) Light intensity distribution obtained by the ordinary microlens array; (**b**) Light intensity distribution obtained by the compound eye.

A square detector of 30 μm × 30 μm was used to analyze the intensity distribution of light. A false color was used as the display form. The result is shown in Figure 6. The distribution of light intensity on the detector corresponding to the microlens array is shown in Figure 6a. The total power displayed on the detector was 0.36372 watts. The light intensity distribution on the detector corresponding to the compound eye is shown in Figure 6b. The total power displayed on the detector was 0.39041 watts. There is still a higher irradiance at the edge of compound eye. The light transmission at the edges of the compound eye is much better than that of the planar microlens array. Obviously, the compound eye structure designed in this paper has a higher utilization of light energy than a planar microlens array.

## 5. Conclusions

The structural design, optical simulation and physical processing of the proposed microlens array were carried out in this paper. The structure of an ordinary microlens array was optimized according to the principle of insect compound eye imaging. A 3D curved compound eye structure composed of a micro-lens array and pinhole array was designed. The microlens array on a curved surface was designed to converge incident lights from different directions onto the imaging plane and improve the utilization of light energy. The pinhole was designed to play a role as a sensitometric beam to effectively reduce the possibility of cross-tracking during imaging and expand the angle of view. The entire compound eye structure has a total of 140 optical channels and a 107.48° × 97.97° theoretical FOV. Femtosecond laser two-photon polymerization technology was adopted to fabricate a bionic compound eye and access a high-precision optical surface. The processed sample was tested by optical profiler. The results showed that the overall size of the structure and the size of the ommatidia were basically consistent with the parameters of our design. An optical system was simulated to investigate the ray tracing and light energy utilization. It can be concluded that the compound eye has a wider field of view and a higher utilization of light energy than the planar microlens array through the results of optical simulation. The research work in this paper may provide a reference value for the structural optimization design of microlens arrays.
